# What can the radiological parameters of superior migration of the humeral head tell us about the reparability of massive rotator cuff tears?

**DOI:** 10.1371/journal.pone.0231843

**Published:** 2020-04-16

**Authors:** Sang-hoon Park, Chong Hyuk Choi, Han-Kook Yoon, Joong-Won Ha, Changmin Lee, Kwangho Chung

**Affiliations:** 1 Department of Orthopaedic Surgery, National Health Insurance Service Ilsan Hospital, Goyang, South Korea; 2 Department of Orthopaedic Surgery, Yonsei University College of Medicine, Seoul, South Korea; 3 Department of Orthopaedic Surgery, Yongin Severance Hospital, Yongin, Gyeonggido, South Korea; Rothman Institute, UNITED STATES

## Abstract

**Objective:**

The interrelation and clinical utility of the parameters for superior migration of the humeral head, such as the acromiohumeral interval (AHI), inferior glenohumeral distance (IGHD), and upward migration index (UMI), in the management of massive rotator cuff tears are not clear. The objectives of this study were to identify the relation between AHI, IGHD, and UMI when measured with radiography and MRI and to determine whether superior migration can predict the irreparability of massive rotator cuff tears.

**Methods:**

We retrospectively reviewed the files of 64 consecutive patients who underwent arthroscopic partial or complete repair for massive rotator cuff tears at our institution between August 2015 and August 2018. We recorded both radiography and MRI measurements of AHI, IGHD, and UMI, and further the tangent sign, fatty infiltration of the rotator cuff muscles, and the Patte grade. We performed correlation assessments and multiple logistic regression analysis to identify potential predictors of the reparability of massive rotator cuff tears.

**Results:**

Thirty-five patients had partially reparable and 29 had completely reparable tears. Parameters measured with either radiography or MRI were highly correlated with each other. The radiographic measurements showed a moderate or low correlation with the MRI measurements. All parameters of superior migration of the humeral head on radiography and MRI, the tangent sign, fatty infiltration of the infraspinatus muscle, and the Patte grade showed significant differences between patients with partially and completely repaired tears. Among these, the independent predictors for irreparability was Patte grade = 3.

**Conclusion:**

The AHI, IGHD, and UMI were highly correlated when measured with either radiography or MRI, but not when comparing their radiographic with their MRI values. Furthermore, they were not independent indicators of reparability in massive rotator cuff tears.

## Introduction

Rotator cuff defects can lead to superior migration of the humeral head, which alters the mechanics of the rotator cuff and results in dysfunction of the shoulder [1]. Proximal migration of the humeral head is one of the key components in the evaluation of rotator cuff disorders. Findings on plain radiographs that have been identified as indicators of rotator cuff tears include a reduced interval between the humeral head and acromion and morphological changes of the greater tuberosity, and humeral neck and head [2–5]. Among those, the acromiohumeral interval (AHI) has been shown to be a sensitive tool for detecting rotator cuff disease since Golding first described it on plain radiographs [3]. The AHI in the normal population has been reported to range from 7 to 14 mm [2,3,6]. A decreased AHI has been found to be associated with rotator cuff tears [3,7], full-thickness rotator cuff tears [8,9], massive rotator cuff tears [1,7], irreparability of rotator cuff tears [10], preoperative impairment of shoulder function [11], high retear rates after surgery [12–14], and fatty degeneration of the rotator cuff [9,15].

Several methods for assessing the migration of the humeral head in rotator cuff tears have been recently investigated. The upward migration index (UMI) on anteroposterior radiographs was found to be an indicator of fatty infiltration of the rotator cuff muscle [16], and an increased inferior glenohumeral distance (IGHD) on magnetic resonance (MR) imaging was shown to be related with irreparability of large and massive rotator cuff tears [17]. While various studies on AHI exist, the literature on the clinical utility of UMI and IGHD is still limited. Furthermore, the relationship between these different parameters of superior migration of the humeral head, when measured with different imaging modalities, and the reparability of massive rotator cuff tears is not clear to date.

We hypothesized that the parameters of superior migration of the humeral head AHI, IGHD, and UMI, are correlated with each other and forecast the surgical reparability in the management of massive rotator cuff tears.

The objectives of this study were to determine whether (1) there is a correlation between AHI, IGHD, and UMI measured on radiographs (XR-AHI, XR-IGHD, and XR-UMI) or MR images (MR-AHI, MR-IGHD, and MR-UMI) and between the radiographic and MRI measurements; and (2) superior migration can predict the reparability of massive rotator cuff tears.

## Materials and methods

### Study population

We retrospectively reviewed the files of 223 consecutive patients with rotator cuff tears who underwent arthroscopic partial or complete repair at our institution between August 2015 and August 2018. Among these, we identified 64 patients who underwent surgery for massive rotator cuff tears. A massive rotator cuff tear was defined as a complete tear involving two or more tendons [18,19]. We excluded patients with (1) a history of surgery of the affected shoulder; (2) apparent glenohumeral arthritis ≥ stage 3 according to the Hamada classification [20]; (3) pseudoparalysis; (4) stiffness; and (5) osseous deformity of the affected shoulder.

An irreparable rotator cuff tear requiring partial repair was defined as a tear that did not allow to completely cover the humeral footprint of rotator cuff muscles at the time of surgery [21]. According to the degree of reparability, we divided patients into two groups: the complete repair group (group C); and the partial repair group (group P).

The Institutional Review Board of National Health Insurance Service Ilsan Hospital (file number: NHIMC 2019-05-003) approved the study and waived the requirement for informed consent.

### Operative technique

All surgical procedures were performed by the same senior surgeon. Patients were positioned in an upright beach-chair position under general anesthesia. We utilized four routine portals: posterior, lateral, anterolateral, and anterior portal. The posterior portal was created first, and the joint was inspected for rotator cuff lesions and other intra-articular lesions. The subacromial space was explored, and the lateral portal was established to evaluate the tear size and configuration. The tear size of the cuff was measured with a probe. The anterolateral portal was placed around the lateral border of the acromion, and the reparability of the torn cuff was assessed through the lateral portal with an arthroscopic grasper. Additional portals were created as necessary to release adhesions and mobilize the tendons.

**Fig 1:**
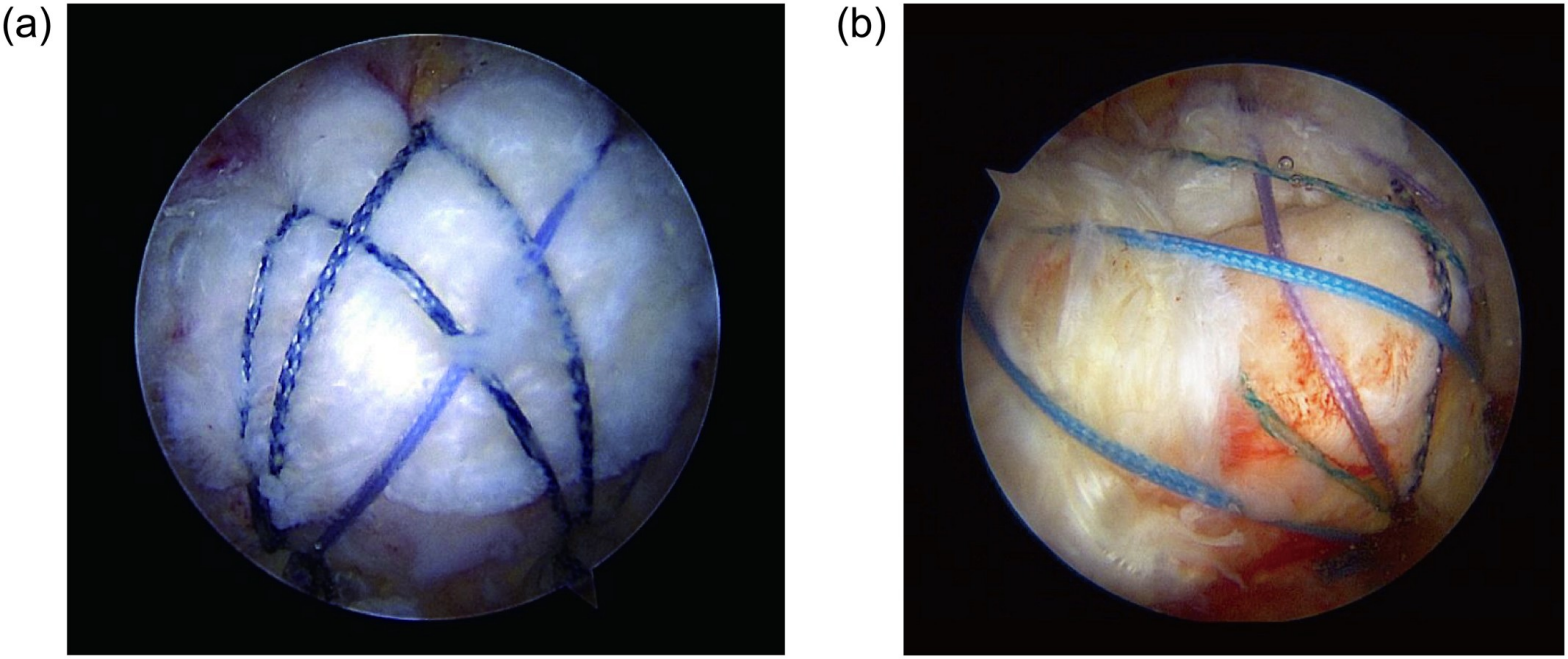
A-B Arthroscopic rotator cuff repair for massive rotator cuff tear. (A) Complete repair with full coverage of the humeral footprint of rotator cuff muscles; (B) Partial repair with incomplete coverage of the footprint.

In patients amenable to a complete arthroscopic repair of their rotator cuff tear (Group C, Fig 1A), we performed either a simple repair or a suture bridge technique, depending on the tear configuration, size, and tissue quality. For the subscapularis tendon, a simple repair was done in a single-row fashion. Patients in whom cuff lesions could not be repaired completely underwent partial arthroscopic repair (Group P, Fig 1B). After sufficient release and mobilization of the cuff muscles, we used the margin convergence technique [22]. Subacromial decompression with removal of bursal tissue and acromioplasty was done in all patients. Biceps tenotomy or tenodesis was performed for symptomatic degenerative biceps lesions involving more than 50% of the tendon according to the age and activity level of the patient.

### Radiographic and MR imaging assessment

At our institution, a preoperative shoulder anterior-posterior (AP) radiograph is obtained for all patients using the same protocol. The shoulder AP radiographs were taken in an upright position with the posterior aspect of the shoulder touching the film cassette and the arm in neutral rotation. The beam was angled 20° craniocaudally, and the film-focus distance was 140 cm. Images were fine-tuned so that the glenohumeral joint was projected without any bony superimposition.

The majority of patients (n = 58) had undergone MR imaging at our institution, and a few patients (n = 8) had done so at other institutions. All MR images were taken with a ≥ 1.5 tesla MR imaging device. All measurements were done electronically with a picture archiving and communication system workstation (Centricity; GE Healthcare, Chicago, Illinois, USA) on radiographs and MR images.

**Fig 2:**
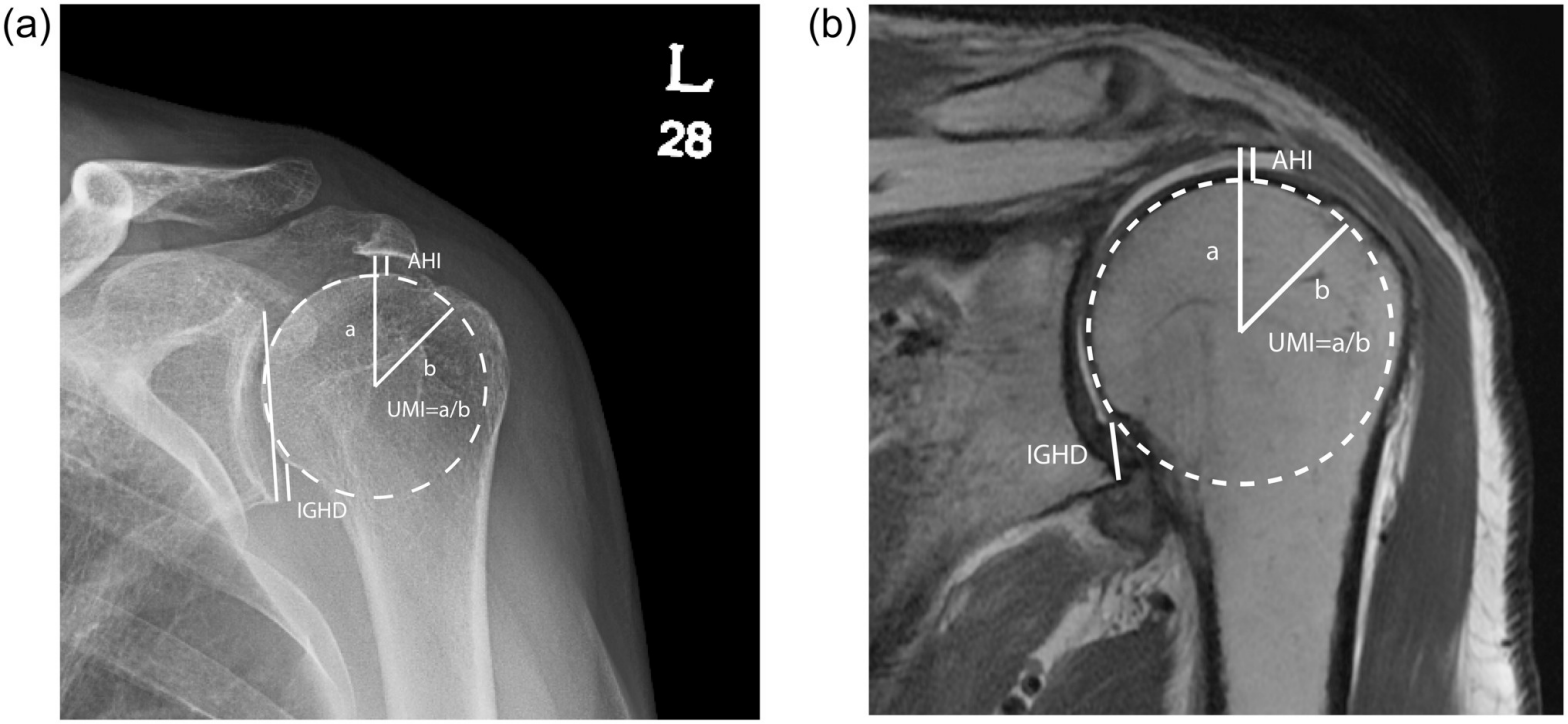
A-B Measurement of the superior humeral migration on anterior-posterior radiograph (A) and coronal magnetic resonance image (B). The acromiohumeral interval (AHI) was defined as the distance between the inferior acromion and the most superior aspect of the humeral head. The inferior glenohumeral distance (IGHD) was measured between the inferior glenoid margin and the inferior humeral head margin, which intersects the humeral head articular margin and the humeral neck. The upward migration index (UMI) was calculated as the distance between the lowest point on the acromion to the center of the humeral head (a), divided by the radius of the humeral head (b).

Three measurements were used to evaluate the upward migration of the humeral head on shoulder AP radiographs: XR-AHI, XR-IGHD, and XR-UMI (Fig 2A), and on MR images in the coronal view: MR-AHI, MR-IGHD, and MR-UMI (Fig 2B). The AHI was defined as the distance between the inferior acromion and the most superior aspect of the humeral head [7]. The IGHD was measured between the inferior glenoid margin and the inferior humeral head margin, which intersects the humeral head articular margin and the humeral neck [17]. The UMI described by Hirooka et al. [23] was calculated as the distance between the lowest point of the acromion and the center of the humeral head, divided by the radius of the humeral head.

To evaluate whether any of these parameters of superior humeral migration may predict the reparability of massive rotator cuff tears, the tangent sign, the degree of tendon retraction, and fatty infiltration of the rotator cuff muscles were recorded and analyzed based on the results of previous studies [10,17,24–26]. The tangent sign was considered positive if the bulk of the supraspinatus muscle failed to touch a line connecting the coracoid process to the scapular spine on sagittal T2-weighted MR images. The extent of the tendon retraction was evaluated using Patte’s classification [27] on coronal T2-weighted MR images. The classification assigns grade 1 when the proximal stump of the tendon is retracted to before the lateral of the articular margin, grade 2 when it is at the level of the humeral head, and grade 3 when it is at the glenoid level. The Goutallier classification [28] was used to measure the degree of fatty infiltration: grade 0 is defined as no fatty deposits, grade 1 as fatty streaks within the muscle tissue, grade 2 as more muscle tissue than fat tissue, grade 3 as comparable amounts of fat and muscle tissue, and grade 4 as more fat than muscle tissue.

Since the cut-off value for the reparability of massive rotator cuff tears in the Patte classification is grade 3, and those of the supraspinatus and infraspinatus fatty infiltrations were grade > 3 and grade > 2, respectively, in a previous study [25], we used theses cut-off values in our analysis. For the fatty infiltration of the subscapularis muscle, we distinguished between ≤ grade 1 and >2 in reference to Kim et al. [10].

Each parameter of superior humeral migration was measured twice with a three-week interval by two blinded investigators separately and randomly to calculate interrater and intrarater reliability. The mean values of the measured variables were used for analysis (S1 Table).

### Statistical analysis

The Shapiro-Wilk test was used to assess normality of data. Pearson’s correlation analysis was used to evaluate a potential interrelation between AHI, IGHD, and UMI on radiographs and MR images. We classified the correlation strength as very high (r = 0.90–1.00), high (r = 0.70–0.89), moderate (r = 0.50–0.69), and low (r = 0.30–0.49) [29]. The student’s t-test was used to compare continuous variables, and the chi-square test to compare categorical variables between group C and group P. The threshold for statistical significance (alpha) was defined as a P-value < 0.05.

Six regression models compared each parameter of superior humeral migration and significantly different variables between group C and group P using the area under the curve (AUC) of the receiver operative curve with DeLong’s method to identify variables for the final regression model while avoiding multicollinearity. Multiple logistic regression analysis using the enter method was applied to identify any factors related to irreparability of massive rotator cuff tears. We used the intraclass correlation coefficient (ICC) to assess interrater and intrarater reliability of measurements. The reliability was categorized as excellent, fair to good, and poor if the ICC was > 0.75, 0.4–0.75, and < 0.4, respectively [30].

We used the IBM SPSS Statistics, version 23.0 (IBM Corp., Armonk, New York, USA) and MedCalc Statistical Software, version 19.2.1 (MedCalc Software, Ostend, Belgium) for the statistical analysis. The statistical power was assessed using G*Power, version 3.1 (Düsseldorf, Nordrhein-Westfalen, Germany) [31].

## Results

### Demographic data and preoperative patient characteristics

Among our 64 patients, 35 patients (54.7%, group P) had rotator cuff tears that were partially reparable and 29 patients (45.3%, group C) had completely reparable tears using arthroscopic procedures. There were no significant differences in gender, age, or the affected side between the two groups (Table 1). All parameters of superior migration of the humeral head on both radiographs and MRI showed significant differences between group C and P. A statistically significant relationship was seen between reparability and the tangent sign, fatty infiltration of the infraspinatus muscle > grade 2, and a Patte grade 3. The number of tendons involved and degree of fatty infiltration of the supraspinatus and subscapularis muscle did not differ between the groups.

**Table 1:** Demographic data and preoperative characteristics of patients (n = 64) with massive rotator cuff tear who underwent complete or partial repair.

Variable	Group C	Group P	P-value
	(n = 29)	(n = 35)	
**Gender**, n (%)			
Male	15 (51.7)	12 (34.3)	0.160
Female	14 (48.3)	23 (65.7)	
**Age** (years), mean ± SD	66.0 ± 6.4	66.3 ± 6.4	0.829
**Affected side**, n (%)			
Right	16 (55.2)	14 (40.0)	0.226
Left	13 (44.8)	21 (60.0)	
**Parameters of superior migration of humeral head** (mm), mean ± SD			
XR-AHI	7.8 ± 1.5	6.6 ± 1.5	0.002
XR-IGHD	6.5 ± 1.8	7.6 ± 1.9	0.015
XR-UMI	1.32 ± 0.08	1.28 ± 0.07	0.042
MR-AHI	6.9 ± 1.4	5.9 ± 1.7	0.014
MR-IGHD	7.8 ± 1.9	8.8 ± 1.9	0.036
MR-UMI	1.33 ± 0.10	1.27 ± 0.08	0.015
**Number of tendons involved**, n (%)			
N ≥ 3	12 (42.9)	19 (54.3)	0.367
N < 3	16 (57.1)	16 (45.7)	
**Tangent sign**, n (%)			
Yes	10 (34.5)	23 (65.7)	0.013
No	19 (65.5)	12 (34.3)	
**Fatty infiltration**, n (%)			
Supraspinatus muscle			
Grade > 3	9 (31.0)	15 (42.9)	0.331
Grade ≤ 3	20 (69.0)	20 (57.1)	
Infraspinatus muscle			
Grade > 2	3 (10.3)	13 (37.1)	0.014
Grade ≤ 2	26 (89.7)	22 (62.9)	
Subscapularis muscle			
Grade > 1	6 (20.7)	12 (34.3)	0.228
Grade ≤ 1	23 (79.33)	23 (65.7)	
**Patte grade**, n (%)			
Grade = 3	10 (34.5)	22 (62.9)	0.024
Grade ≤2	19 (65.5)	13 (37.1)	

P: partial repair; C: complete repair; SD: standard deviation; XR-AHI: Acromiohumeral interval on radiograph; XR-IGHD: inferior glenohumeral distance on radiograph; XR-UMI: upward migration index on radiograph; MR-AHI: Acromiohumeral interval on MRI; MR-IGHD: inferior glenohumeral distance on MRI; MR-UMI: upward migration index on MRI.

### Inter-relationship between the superior humeral migration parameters

The Pearson correlation coefficients (r) for the different parameters of the superior migration of the humeral head are shown in Table 2. The radiographically measured parameters were highly correlated with each other, and so were all parameters measured on MRI. The radiographic parameters showed a moderate correlation with the MRI parameters except for XR-AHI and MR-IGHD that showed a low correlation.

**Table 2:** Interrelation of the different parameters of superior migration of humeral head in 64 patients with massive rotator cuff tears.

	Correlation coefficient (r)					
Variable	XR-AHI	XR-IGHD	XR-UMI	MR-AHI	MR-IGHD	MR-UMI
XR-AHI		-0.720**	0.801**	0.616**	-0.456**	0.569**
XR-IGHD			-0.713**	-0.517**	0.632**	-0.501**
XR-UMI				0.510**	-0.515**	0.505**
MR-AHI					-0.779**	0.842**
MR-IGHD						-0.777**

** The correlation is significant at P-value < 0.001 level.

XR-AHI: Acromiohumeral interval on radiograph; XR-IGHD: inferior glenohumeral distance on radiograph; XR-UMI: upward migration index on radiograph; MR-AHI: Acromiohumeral interval on MRI; MR-IGHD: inferior glenohumeral distance on MRI; MR-UMI: upward migration index on MRI.

The intraclass correlation coefficients for inter-rater reliability of the measurement of parameters were excellent (ICC > 0.75). The intraclass correlation coefficients for intrarater reliability were excellent (ICC > 0.75) for investigator 1 and 2.

### Prediction of irreparability

The results of the six multiple logistic regression models for the significant variables are shown in the S2–S7 Tables. Among the parameters of superior humeral migration, XR-AHI, MR-AHI, and MR-UMI were statistically significant for predicting irreparability. Since MR-AHI and MR-UMI were highly linearly correlated with each other (r = 0.842), and the regression models for MR-AHI (AUC = 0.785) and MR-UMI (AUC = 0.788) were comparable (P = 0.907), we decided to use MR-AHI as the variable for the final regression model. XR-AHI and MR-AHI were related, but the correlation was not high. Therefore, we used MR-AHI as another variable for the final regression model. Among the different variables, only a Patte grade = 3 was an independent predictor of reparability (Table 3). The statistical power using Patte classification of the final regression model calculated using G*Power was 89.5%.

**Table 3:** Multiple regression predicting irreparability of massive rotator cuff tears (n = 64).

Variable	Estimate	Standard error	Odds ratio	95% Confidence interval	P-value
XR-AHI	-0.361	0.252	0.697	0.426–1.142	0.152
MR-AHI	-0.264	0.251	0.768	0.469–1.257	0.294
Tangent sign	0.510	0.637	1.665	0.478–5.799	0.423
Fatty infiltration of IST > grade 2	0.967	0.814	2.631	0.534–12.965	0.235
Patte grade 3	1.414	0.629	4.111	1.198–14.108	0.025

XR-AHI: Acromiohumeral interval on radiograph; MR-AHI: Acromiohumeral interval on MRI; IST: infraspinatus tendon.

## Discussion

The superior migration of the humeral head is known as a secondary phenomenon of progressive rotator cuff disease resulting from an imbalance between the force couple of the rotator cuff muscles [26]. Consequently, methods to quantitatively measure superior humeral migration and assess their clinical usefulness have been developed so far. There are several ways to determine the degree of the superior migration of the humeral head, including AHI, IGHD, and UMI. However, few studies have investigated the relationship between these parameters of superior humeral migration on the one hand and their measurement with different imaging methods on the other hand. Furthermore, while some recent studies evaluated the utility of superior migration indexes as predictors of the reparability of rotator cuff tears [10,17,26,32], no study has done this comprehensively and exclusively for massive rotator cuff tears. The present study was focused on investigating the interrelationship between the superior humeral migration indexes AHI, IGHD, and UMI per se and when measured using different imaging modalities on the one hand, and their capability to predict the reparability of massive rotator cuff tears on the other hand.

Our results show that there is a high correlation between the different parameters of superior humeral migration when measured with either radiography or MR imaging, but not when comparing the measurements between the two imaging modalities. The parameters may compensate for each other when evaluating the superior humeral migration within one imaging modality and replace each other when one parameter is not measurable because of pathoantomical changes. Patients with an irreparable massive rotator cuff tear treated with partial repair had a significantly lower AHI and UMI and higher IGHD on radiographs and MRI compared to patients with completely reparable tears. There was a significant difference in the presence of the tangent sign, a Patte grade 3, fatty infraspinatus infiltration > grade 2, and in the values for AHI, IGHD, and UMI on either radiography or MR images. However, the indices of superior migration of the humeral head could not predict the irreparability of massive rotator cuff tears in our patients.

Previous studies have shown that the measurement of superior migration of the humeral head using standardized protocols for radiography and MR imaging is critical to ensure interrater reliability and reproducibility. Gruber et al. [33] showed that the interrater reliability of radiographic AHI assessments by five independent physicians was high when using standardized radiographs. Contrary to that, Bernhardt et al. [34] demonstrated that the measurement of the AHI by orthopedic surgeons using non-standardized radiographs was neither reliable nor reproducible. Therefore, we only used standardized standing anterior-posterior radiographs and supine MR imagines of the shoulder in our study. This is rational because these methods are routinely used in our clinic as a preoperative evaluation of rotator cuff tears. The inter- and intrarater reliability was high for the parameters in our study.

A few studies compared the parameters of superior migration of the humeral head measured with different imaging modalities. Van de Sande and Rozing [35] found a high correlation between computed tomography and standardized supine radiographs for the measurement of the UMI. They concluded that the UMI is a reliable and accurate parameter for the proximal migration of the humeral head. However, a standing shoulder radiograph, as in our study, is more common than a supine shoulder radiograph. We found a moderate correlation between the UMI measurements obtained with the two imaging modalities, probably because of the body position when taking the radiograph. Werner et al. [36] showed a moderate correlation between the AHI measurements using conventional radiography or MR imaging and emphasized that the MR values were smaller than the corresponding radiographic values. These discrepancies between the indices when using different imaging modalities correspond to our findings and might be attributed to the positioning of the patient and a resulting difference in muscle tension. Since the radiograph is taken upright, while the MR images are obtained in the supine position, gravity could influence the measured values [36,37]. In our study, the mean value of the MR-AHI (6.4 ± 1.6 mm) was smaller than that of the XR-AHI (7.2 ± 1.6 mm). Likewise, the mean value of MR-IGHD (8.4 ± 2.0 mm) was larger than that of XR-IGHD (7.1 ± 1.9 mm).

The anatomical restoration of the tendon to the anatomic footprint is a goal in rotator cuff tear repair. However, complete repair may be difficult in massive rotator cuff tears because of tendon retraction and poor tissue quality [17,18]. The rate of irreparable rotator cuff tears has been reported to range from 6.5% to 30% [36,38–40]. Kim et al. [25] even reported a rate as high as 52% for massive rotator cuff tears. Several studies evaluated the degree of the superior migration of the humeral head as one of the possible indicators for the reparability of a rotator cuff tear. Dwyer et al. [17] found that the IGHD on MRI, but not the AHI, was related to the reparability of large-to-massive rotator cuff tears. However, this finding was not significant in their multivariable analysis. Kim et al. [26] developed a scoring system to predict whether a large-to-massive rotator cuff tear can be repaired based on a retrospective review of patients who had undergone complete or incomplete repair. In that study, the IGHD was not included as an independent predictor, because it was not significant when considered other factors. More recently, Kim et al. [10] showed that the AHI measured on MR imaging was an independent predictor of irreparability in large-to-massive rotator cuff tears. Although the parameters of superior humeral migration were significantly different between the complete and partial repair group in our study, none of the indices of superior humeral migration was an independent predictor of irreparability in massive rotator cuff tear. A possible explanation for the different results is that Kim et al. [10] investigated large-to-massive rotator cuff tears, while we focused on massive tears only. We conclude that the parameters of superior migration of the humeral head are correlated with complete arthroscopic reparability of massive rotator cuff tears, but neither of them alone can predict the reparability. Individual parameters seem less powerful, whereas their combination or other possible indicators may predict the reparability more accurately.

Our study has several limitations. First, because of its retrospective nature and small sample size, our results cannot be generalized. Second, measurement bias could have been present since only two orthopedic surgeons performed all measurements. Third, we only considered the parameters of superior humeral migration, fatty infiltration of the rotator cuff muscles, tangent sign, and degree of tendon retraction in our study. Even though these factors are known to have a predictive value for the reparability of a rotator cuff tear, there could be other factors that influence reparability.

## Conclusions

The parameters of superior humeral migration AHI, IGHD, and UMI are highly correlated with each other when measured either with radiography or MRI, but not when comparing their measurements between the two modalities. None of the parameters of superior migration of humeral head was an independent indicator of the irreparability of massive rotator cuff tears.

## Supporting information

S1 Table Patient data. (XLSX)

S2 Table Multiple logistic regression model 1. (DOCX)

S3 Table Multiple logistic regression model 2. (DOCX)

S4 Table Multiple logistic regression model 3. (DOCX)

S5 Table Multiple logistic regression model 4. (DOCX)

S6 Table Multiple logistic regression model 5. (DOCX)

S7 Table Multiple logistic regression model 6. (DOCX)
