# Supplementary material for: What can the radiological parameters of superior migration of the humeral head tell us about the reparability of massive rotator cuff tears?
Source: PLoS One. 2020 Apr 16;15(4):e0231843. doi: 10.1371/journal.pone.0231843 (PMC7162485; doi:10.1371/journal.pone.0231843)
Supplement: S2 Table — (DOCX) [file pone.0231843.s002.docx]

**S2 Table. Multiple logistic regression model 1**

| **Variable** | **Estimate** | **Standard error** | **Odds ratio** | **95% Confidence interval** | **P-value** |
| --- | --- | --- | --- | --- | --- |
| XR-AHI | -0.499 | 0.221 | 0.607 | 0.394-0.935 | 0.024 |
| Tangent sign | 0.624 | 0.620 | 1.866 | 0.553-6.294 | 0.314 |
| Fatty infiltration of IST > grade 2 | 0.848 | 0.796 | 2.336 | 0.491-11.115 | 0.286 |
| Patte grade 3 | 1.314 | 0.608 | 3.722 | 1.131-12.248 | 0.031 |

XR-AHI: Acromio-humeral interval on radiograph
