# Supplementary material for: What can the radiological parameters of superior migration of the humeral head tell us about the reparability of massive rotator cuff tears?
Source: PLoS One. 2020 Apr 16;15(4):e0231843. doi: 10.1371/journal.pone.0231843 (PMC7162485; doi:10.1371/journal.pone.0231843)
Supplement: S3 Table — (DOCX) [file pone.0231843.s003.docx]

**S3 Table. Multiple logistic regression model 2**

| **Variable** | **Estimate** | **Standard error** | **Odds ratio** | **95% Confidence interval** | **P-value** |
| --- | --- | --- | --- | --- | --- |
| XR-IGHD | 0.270 | 0.159 | 1.310 | 0.960-1.788 | 0.089 |
| Tangent sign | 0.724 | 0.609 | 2.063 | 0.626-6.803 | 0.234 |
| Fatty infiltration of IST > grade 2 | 1.028 | 0.786 | 2.795 | 0.599-13.033 | 0.191 |
| Patte grade 3 | 1.159 | 0.578 | 3.188 | 1.027-9.897 | 0.045 |

XR-IGHD: inferior glenohumeral distance on radiograph
