# Supplementary material for: What can the radiological parameters of superior migration of the humeral head tell us about the reparability of massive rotator cuff tears?
Source: PLoS One. 2020 Apr 16;15(4):e0231843. doi: 10.1371/journal.pone.0231843 (PMC7162485; doi:10.1371/journal.pone.0231843)
Supplement: S4 Table — (DOCX) [file pone.0231843.s004.docx]

**S4 Table. Multiple logistic regression model 3**

| **Variable** | **Estimate** | **Standard error** | **Odds ratio** | **95% Confidence interval** | **P-value** |
| --- | --- | --- | --- | --- | --- |
| XR-UMI | -5.017 | 4.021 | 0.007 | 0.000-17.537 | 0.212 |
| Tangent sign | 0.738 | 0.614 | 2.091 | 0.628-6.964 | 0.229 |
| Fatty infiltration of IST > grade 2 | 1.046 | 0.773 | 2.847 | 0.625-12.960 | 0.176 |
| Patte grade 3 | 1.152 | 0.571 | 3.165 | 1.033-9.696 | 0.044 |

XR-UMI: upward migration index on radiograph
