# Supplementary material for: What can the radiological parameters of superior migration of the humeral head tell us about the reparability of massive rotator cuff tears?
Source: PLoS One. 2020 Apr 16;15(4):e0231843. doi: 10.1371/journal.pone.0231843 (PMC7162485; doi:10.1371/journal.pone.0231843)
Supplement: S5 Table — (DOCX) [file pone.0231843.s005.docx]

**S5 Table. Multiple logistic regression model 4**

| **Variable** | **Estimate** | **Standard error** | **Odds ratio** | **95% Confidence interval** | **P-value** |
| --- | --- | --- | --- | --- | --- |
| MR-AHI | -0.454 | 0.221 | 0.635 | 0.412-0.980 | 0.040 |
| Tangent sign | 0.634 | 0.620 | 1.885 | 0.560-6.350 | 0.306 |
| Fatty infiltration of IST > grade 2 | 1.167 | 0.792 | 3.211 | 0.680-15.154 | 0.141 |
| Patte grade 3 | 1.354 | 0.610 | 3.873 | 1.171-12.807 | 0.026 |

MR-AHI: Acromio-humeral interval on MRI
