# Supplementary material for: What can the radiological parameters of superior migration of the humeral head tell us about the reparability of massive rotator cuff tears?
Source: PLoS One. 2020 Apr 16;15(4):e0231843. doi: 10.1371/journal.pone.0231843 (PMC7162485; doi:10.1371/journal.pone.0231843)
Supplement: S6 Table — (DOCX) [file pone.0231843.s006.docx]

**S6 Table. Multiple logistic regression model 5**

| **Variable** | **Estimate** | **Standard error** | **Odds ratio** | **95% Confidence interval** | **P-value** |
| --- | --- | --- | --- | --- | --- |
| MR-IGHD | 0.310 | 0.163 | 1.364 | 0.991-1.877 | 0.057 |
| Tangent sign | 0.628 | 0.617 | 1.874 | 0.560-6.274 | 0.308 |
| Fatty infiltration of IST > grade 2 | 1.323 | 0.803 | 3.755 | 0.779-18.108 | 0.099 |
| Patte grade 3 | 1.291 | 0.594 | 3.637 | 1.136-11.648 | 0.030 |

MR-IGHD: inferior glenohumeral distance on MRI
