# Supplementary material for: What can the radiological parameters of superior migration of the humeral head tell us about the reparability of massive rotator cuff tears?
Source: PLoS One. 2020 Apr 16;15(4):e0231843. doi: 10.1371/journal.pone.0231843 (PMC7162485; doi:10.1371/journal.pone.0231843)
Supplement: S7 Table — (DOCX) [file pone.0231843.s007.docx]

**S7 Table. Multiple logistic regression model 6**

| **Variable** | **Estimate** | **Standard error** | **Odds ratio** | **95% Confidence interval** | **P-value** |
| --- | --- | --- | --- | --- | --- |
| MR-UMI | -7.666 | 3.580 | 0.000 | 0.000-0.522 | 0.032 |
| Tangent sign | 0.611 | 0.623 | 1.843 | 0.543-6.250 | 0.327 |
| Fatty infiltration of IST > grade 2 | 1.265 | 0.797 | 3.544 | 0.743-16.900 | 0.112 |
| Patte grade 3 | 1.301 | 0.603 | 3.674 | 1.127-11.977 | 0.031 |

MR-UMI: upward migration index on MRI;
